# Mechanical Stress Triggers Cardiomyocyte Autophagy through Angiotensin II Type 1 Receptor-Mediated p38MAP Kinase Independently of Angiotensin II

**DOI:** 10.1371/journal.pone.0089629

**Published:** 2014-02-21

**Authors:** Li Lin, Chuyi Tang, Jianfeng Xu, Yong Ye, Liqing Weng, Wei Wei, Junbo Ge, Xuebo Liu, Yunzeng Zou

**Affiliations:** 1 Department of Cardiovascular Medicine, East Hospital, Tongji University School of Medicine, Shanghai, China; 2 Shanghai Institute of Cardiovascular Diseases, Zhongshan Hospital and Institute of Biomedical Science, Fudan University, Shanghai, China; University of Western Ontario, Canada

## Abstract

Angiotensin II (Ang II) type 1 (AT_1_) receptor is known to mediate a variety of physiological actions of Ang II including autophagy. However, the role of AT_1_ receptor in cardiomyocyte autophagy triggered by mechanical stress still remains elusive. The aim of this study was therefore to examine whether and how AT_1_ receptor participates in cardiomyocyte autophagy induced by mechanical stresses. A 48-hour mechanical stretch and a 4-week transverse aorta constriction (TAC) were imposed to cultured cardiomyocytes of neonatal rats and adult male C57B/L6 mice, respectively, to induce cardiomyocyte hypertrophy prior to the assessment of cardiomyocyte autophagy using LC3b-II. Losartan, an AT_1_ receptor blocker, but not PD123319, the AT_2_ inhibitor, was found to significantly reduce mechanical stretch-induced LC3b-II upregulation. Moreover, inhibition of p38MAP kinase attenuated not only mechanical stretch-induced cardiomyocyte hypertrophy but also autophagy. To the contrary, inhibition of ERK and JNK suppressed cardiac hypertrophy but not autophagy. Intriguingly, mechanical stretch-induced autophagy was significantly inhibited by Losartan in the absence of Ang II. Taken together, our results indicate that mechanical stress triggers cardiomyocyte autophagy through AT_1_ receptor-mediated activation of p38MAP kinase independently of Ang II.

## Introduction

Under physiological conditions, cardiac hypertrophy serves as a compensatory response to increased hemodynamic load, including hypertension [1], vascular disease [2], and myocardial infarction [3]. However, sustained or excessive hypertrophic responses may prompt transition to decompensated pathological hypertrophy *en route* to ultimate heart failure [4]. The definite irreversible cellular sequelae for decompensated cardiac hypertrophy is cardiomyocyte death, as evidenced by loss of cardiomyocyte histologically [5]. Three distinct modes of cell death have been demonstrated including necrosis (oncosis), apoptosis, and autophagy [6]. Necrosis and apoptosis invariably contribute to cell death, whereas autophagy may serve as a double-edged sword to provide both pro-survival and pro-death roles. In patients with heart failure, autophagy was found to be excessive, much more than that of apoptosis, correlating with left ventricular systolic dysfunction [6], [7].

Autophagy is a highly conserved catabolic cellular process for protein and nutrient recycling. Initially described as a cellular survival mechanism in starvation, autophagy has recently gained much attention as a mechanism for cell death in a number of diseases including heart failure [8]. Excessive autophagy has been documented in myocardial damages, contributing to the onset and progression of pathological conditions such as pressure overload-induced cardiac remodeling and heart failure [9], [10], [11]. Angiotensin II (AngII) and its type 1 receptor (AT_1_) have been reported to play a pivotal role in the etiology of heart failure possibly related to autophagy [12]. Nonetheless, the precise role of AT_1_ receptor and autophagy, if any, in pressure overload-induced heart failure remains elusive. Moreover, the mitogen-activated protein kinase (MAPK) family such as ERKs, JNK and p38MAP kinase have been implicated in pressure overload-induced cardiac hypertrophy [13], [14]. Nonetheless, whether these stress signaling molecules are involved in cardiomyocyte autophagy remains elusive. To this end, the present study was designed to examine the role of AT_1_ receptor in pressure overload-induced cardiomyocyte autophagy en route to heart failure, and the role of ERKs, JNK and p38MAP kinase in AT_1_ receptor-mediated myocardial autophagy responses, if any.

## Methods

### Animal models

All animal procedures were approved by the Animal Care and Use Committee of Fudan University and were in compliance with the Guidelines for the Care and Use of Laboratory Animals published by the National Academy Press (NIH Publication No. 85-23, revised in 1996). In brief, C57BL/6 male mice, aged 8–10 weeks, were purchased from the Jackson Laboratory (Bar Harbor, Maine, USA) and were subjected to transverse aorta constriction (TAC) or sham operation under anesthesia (ketamine, 25 mg/kg, i.p.) as described [15]. Following anesthetization and artificial ventilation, the transverse aorta was constricted with the 7-0 nylon suture by ligating the aorta together using a blunted 27-gauge needle. The needle was pulled out immediately after the procedure. Losartan (3 mg/kg/day, Sigma-Aldrich), SB203580 (10 mg/kg/day, Sigma-Aldrich) or PBS (used as a vehicle) was continuously administered using Alzet osmotic minipumps (Model 2002, DURECT, Cupertino) implanted subcutaneously into the back of mice 3 days prior to TAC. Four weeks later, all mice were sacrificed and hearts were excised for further examination.

### Echocardiography and haemodynamic measurements

Transthoracic echocardiography was performed using 30MHz high frequency scanhead (VisualSonics Vevo770, VisualSonics Inc. Toronto, Canada) [16]. All measurements, averaged for five consecutive cardiac cycles, were carried out by three experienced technicians unaware of experimental group identity. Blood pressure (BP) was evaluated as described [17], [18]. A micronanometer catheter (Millar 1.4F, SPR 835, Millar Instruments, Inc., Houston, TX) was inserted into the right common carotid artery, while the transducer was connected to a Power Laboratory system (AD Instruments, Castle Hill, Australia) in order to record BP.

### Morphology and histological Analyses

Excised hearts were weighed, perfused with PBS and fixed with 4% polyformaldehyde for global morphometry and then with 10% formalin for further histological analysis. Paraffin-embedded hearts were sectioned at 4-μm thickness and stained with hematoxylin and eosin (H-E). Cardiomyocyte morphology and histology was visualized under a high magnification to assess cross-sectional area (CSA) using a video camera (Leica Qwin 3) attached to a micrometer. Twenty randomly chosen fields were evaluated from each cross section of the left ventricle (LV) free wall.

### Cell culture and treatment

Primary cultured cardiomyocytes derived from neonatal rats [19](Shanghai Institutes for Biological Sciences, Shanghai, China) and COS7 cells [20](Shanghai Institutes for Biological Sciences, Shanghai, China) were cultured in Dulbecco's modified Eagle's medium (DMEM) with 10% fetal bovine serum (FBS). The cells were then incubated in serum and antibiotic-free conditions in silicon-based plates pre-coated for 24 hours with collagen prior to application of 48-hour mechanical stretch to 120% [15]. Losartan (10^−6^ mol/L), PD123319 (10^−6^ mol/L), PD98059 (10^−5^ mol/L), SB203580 (10^−5^ mol/L), or SP600125 (10^−5^ mol/L) was pre-administered in the culture medium for 30 min. The mechanical stretch last for 48 hours and cardiomyocytes were collected for extraction of protein and total RNA for further study. For transfection experiments, COS7 cells were incubated for 24 hours before transfection.

### [^3^H] Leucine Incorporation

Cultured cardiomyocytes were incubated with [^3^H] leucine (1 µCi/ml) in silicon-based plates pre-coated with collagen. Cells were then subjected to a 48-hour mechanical stretch before being exposed with 5% trichloroacetic acid. Protein precipitates were dissolved in 1 ml of 100 mmol/L NaOH and radio activity was determined using a liquid scintillation counter.

### Real-time RT-PCR

Total RNA was isolated from LV tissues or culture cells using TRIZol® reagent according to the manufacturer's instruction. After purification, RNA was subjected to real-time RT-PCR analysis for the expression of atrial natriuretic peptide (*Anp*) and skeletal α-actin (*Saa*) on a BIO-RAD IQ5 multicolor detection system. The melting curves and quantization were analyzed using Light Cycler and Rel Quant software, respectively. A comparative CT method was used to determine relative quantification of RNA expression [21]. All PCR reactions were performed at least in triplicate.

### Western blot analyses

Total proteins isolated from LV tissues or culture cells were detected for LC3b-II using western blot analysis. Cells were lysed before centrifuged at 200×*g* to remove the nuclei. The supernatant was centrifuged at 15,000×*g* for 30 min to collect cell membrane. Total proteins were size-fractionated by SDS-PAGE and were transferred to Immobilon-P membranes (Millipore). The blotted membranes were incubated with antibodies against LC3b-II (Cell Signaling Technology Inc. Beverly, MA, USA), and subjected to an ECL Detection system (GE healthcare).

### Autophagic flux assessment

For autophagic flux assessment, cardiomyocytes were treated with vehicle or chloroquine (CQ, 20umol/L) [22] for 4 hours and cell lysates were prepared for the detection of LC3b-II protein abundance.

### Generation of GFP-LC3 vector and fluorescent microscopic analysis

A lentiviral vector containing GFP-LC3 reporter (GFP-LC3) was constructed. Cardiomyocytes were transfected with lentivirus particles (MOI = 20) and then were mechanical stretched for 48 hours. Cells were observed under fluorescent microscope.

### Immunofluorescence

Following cell culture on silicon-based plates in serum-free DMEM for 48 hours, cardiomyocytes or COS7 cells were incubated with anti-α-MHC (Upstate, catalogue 05-716, USA) and LC3b-II (Cell Signaling Technology Inc. catalogue 2775, USA). The samples were then incubated with secondary antibodies conjugated with FITC or Alex (Invitrogen, catalogue A21206, USA) according to the manufacturer's instructions. The surface areas of cardiomyocytes and LC3b-II were determined using an image analysis software (Leica Qwin 3) and were calculated using the mean of 100 to 120 cells from randomly selected fields.

### Statistical analysis

Data are shown as means ± s.e.m. Comparison was performed by one-way analysis of variance followed by Newman-Keuls test for post-hoc analysis to determine the difference among the groups.

## Results

### 1. Involvement of AT_1_ receptor in mechanical stretch-induced cardiomyocyte hypertrophic and autophagic responses

**Figure 1:**
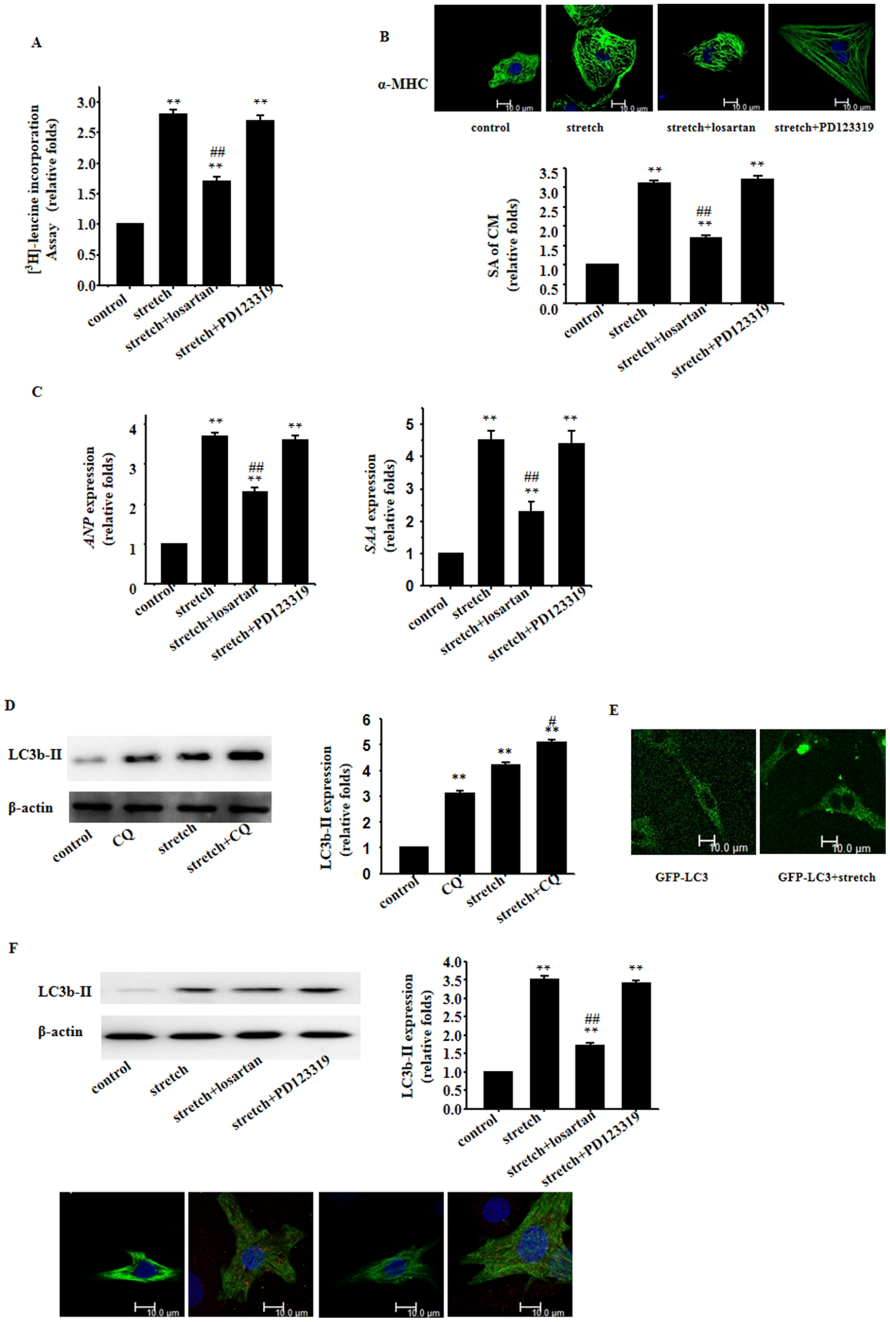
Involvement of AT_1_ receptor in mechanical stretch-induced cardiomyocyte hypertrophic and autophagic responses. Cultured cardiomyocytes of neonatal rats were treated with vehicle (Control), stretch in the absence or presence of Losartan (10^−6^ mol/L) or PD123319 (10^−6^ mol/L); **A**: [^3^H]-Leucine incorporation in cardiomyocytes; **B**: Cardiomyocyte morphology and size; cardiomyocytes were subjected to immunofluorescent staining for α-MHC (green) and DAPI; Representative photographs were shown from 3 independent experiments (scale bar: 10 µm); surface area (SA) of cardiomyocytes was evaluated by measuring 100 cardiomyocytes from each dish; **C**: Expressions of *Anp and Saa* genes evaluated by real time RT- PCR; *β-Actin* serving as the internal control; **D**: Western blot analyses for LC3b-II using the anti LC3b-II antibody; β-actin in whole cell lysate was used as the loading control; **E**: Fluorescence microscopy analysis for GFP-LC3; **F**: Western blot analyses and immunofluorescent staining for LC3b-II using the anti LC3b-II antibody (red); β-actin in whole cell lysate was used as the loading control. Representative photograms from 3 independent experiments are shown. All data are expressed as mean ± S.E.M from 3 independent experiments, ^**^ *p*<0.01 versus cardiomyocytes in the control; ^##^ *p*<0.01 versus cardiomyocytes with stretch.

Mechanical stretch-induced hypertrophic responses were manifested as increased cellular protein synthesis in neonatal cardiomyocytes as evaluated by the [^3^H]-Leucine incorporation assay (Fig. 1A), enlarged cell surface area measured by immunofluorescence (Fig. 1B), and reprogramming of hypertrophy-associated fetal genes including *Anp* and *Saa* measured using quantitative real-time PCR (Fig. 1C). Interestingly, the mechanical stress-induced hypertrophic responses were significantly attenuated by treatment with the AT_1_ receptor antagonist Losartan (Fig. 1A–C). However, treatment with the AT_2_ receptor antagonist PD123319 failed to affect mechanical stretch-induced hypertrophic responses (Fig. 1 A–C).

Cultured cardiomyocytes exhibit a significant increase in cardiomyocyte autophagy as evidenced by the increased LC3b-II following a 48-hour mechanical stretch (Fig. 1D). To examine whether mechanical stretch affects autophagic flux, we subjected cardiomyocytes with mechanical stretch in the absence and presence of chloroquine, which inhibits lysosomal acidification and prevents autophagosome-lysosome fusion. The control group demonstrated a basal level of cardiomyocyte autophagy, with autophagosome accumulation (≈3-fold increase) in the presence of chloroquine, suggesting the intact autophagic flux. Mechanical stretch caused a 5.1-fold increase in autophagosome abundance compared with the control group, implying the induction of autophagosome formation (Fig. 1D). To confirm the characteristics of autophagy elicited by mechanical stress, puncta GFP-LC3 and accumulation of autophagosomes in cardiomyocytes were investigated by fluorescence microscopy. Fluorescence microscopy analysis showed that a punctate pattern of LC3 was observed in mechanical stress-treated cardiomyocytes, whereas a diffused distribution of LC3 was found in control cardiomyocytes (Fig. 1E). To ask whether AT1 or AT2 receptor was involved in mechanical stretch-induced cardiomyocyte autophagy, we used pharmacological inhibitors. Intriguingly, the AT1 receptor blocker Losartan, but not AT2 receptor inhibitor PD123319, drastically suppressed mechanical stretch-induced cardiomyocyte autophagy (Fig. 1F).

### 2. Involvement of p38MAP kinase in mechanical stretch-induced cardiomyocyte autophagy

**Figure 2:**
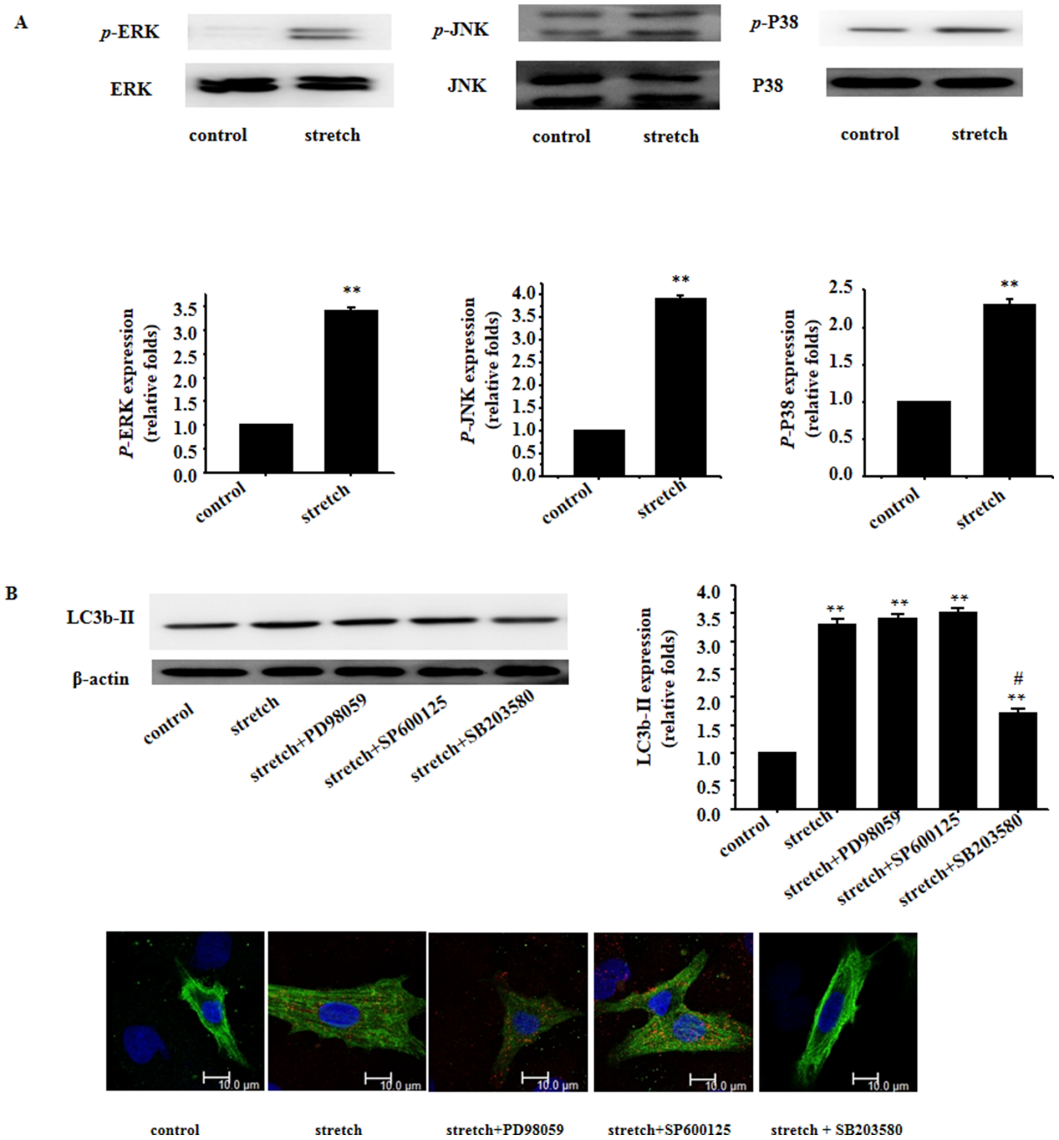
Involvement of p38MAP kinase in mechanical stretch-induced cardiomyocyte autophagy. **A**: Phosphorylation of ERKs, JNK and p38MAP kinase was examined using Western blotting; Total protein levels of ERKs, JNK and p38MAP kinase were used as the loading control. Representative photograms were shown from 3 independent experiments; **B**: Western blot analyses and immunofluorescent staining for LC3b-II using the anti LC3b-II antibody (red); β-actin in whole cell lysate was used as the loading control. Representative photograms from 3 independent experiments are shown.

To examine whether the MAPK family participates in mechanical stress-induced cardiac autophagy, phosphorylation of ERKs, JNK and p38MAP kinase were evaluated. Our results revealed significantly elevated phosphorylation of these stress signaling molecules in response to mechanical stretch (Fig. 2A). Although inhibition of ERKs, JNK and p38MAP kinase all ablated mechanical stress-induced cardiac hypertrophic responses, only p38 MAPK inhibitors but not ERK or JNK suppressed mechanical stretch-induced autophagic responses (Fig. 2B).

### 3. Mechanical stress-induced autophagy through AT_1_ receptor and p38MAP kinase without Ang II

**Figure 3:**
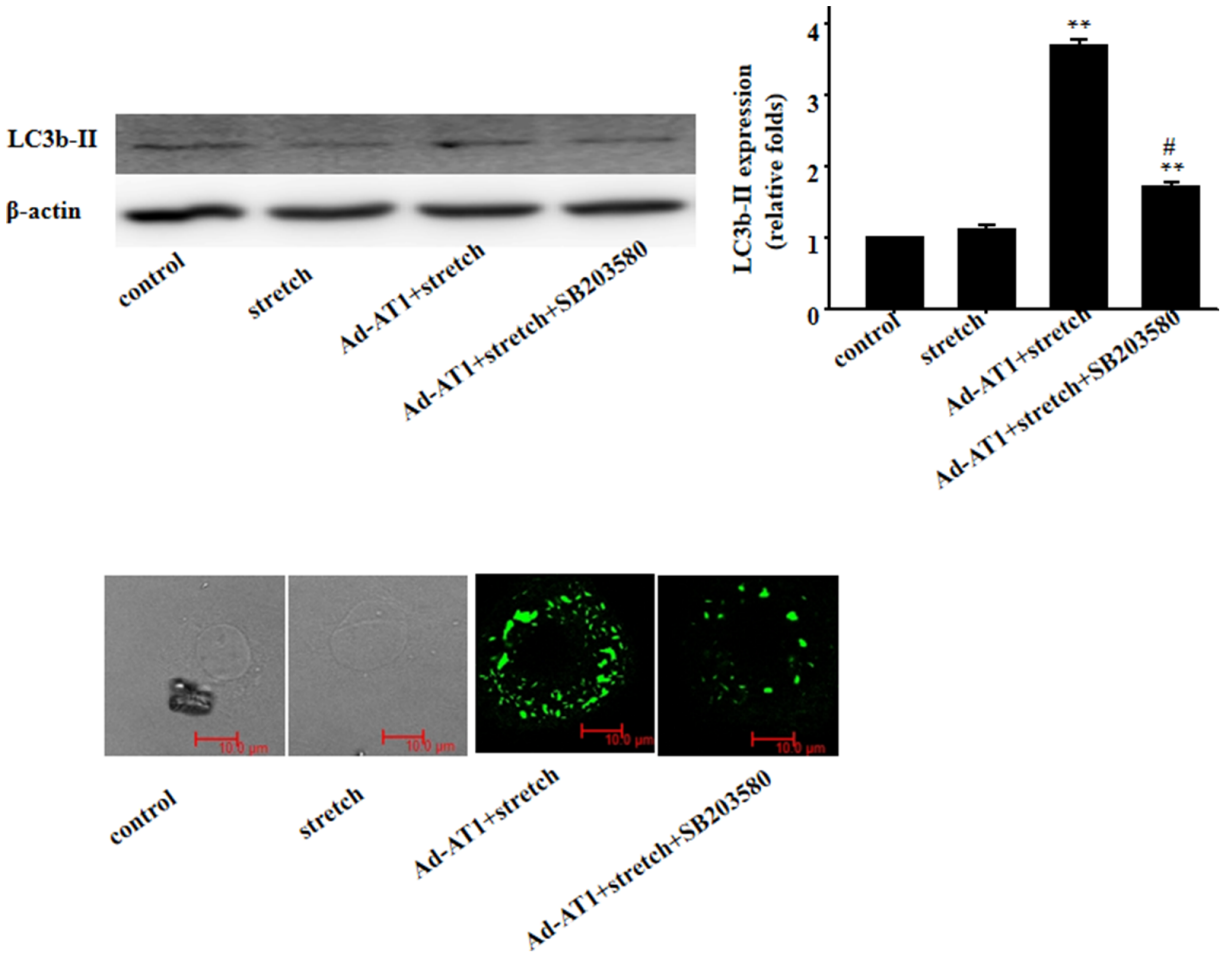
Mechanical stress induced autophagy through AT_1_ and p38MAP kinase independently of Ang II. Western blot analyses and immunofluorescent staining for LC3b-II using specific antibody (green); β-actin in whole cell lysate was used as loading control. Representative photograms from 3 independent experiments are shown.

To examine if mechanical stress induces autophagy through AT_1_ receptor independent of Ang II, we examined COS7 cells exposed to mechanical stress. Our data revealed that stimulated COS7 cells failed to induce a significant increase in autophagy (as evidenced by LC3b-II) in response to mechanical stress. However, they developed an increase in autophagy when transfected with AT_1_ receptor. Moreover, mechanical stress-induced autophagy was suppressed by p38MAP kinase inhibitor (Fig. 3).

### 4. Involvement of cardiomyocyte autophagy in pressure overload-induced cardiac hypertrophy and dysfunction

**Figure 4:**
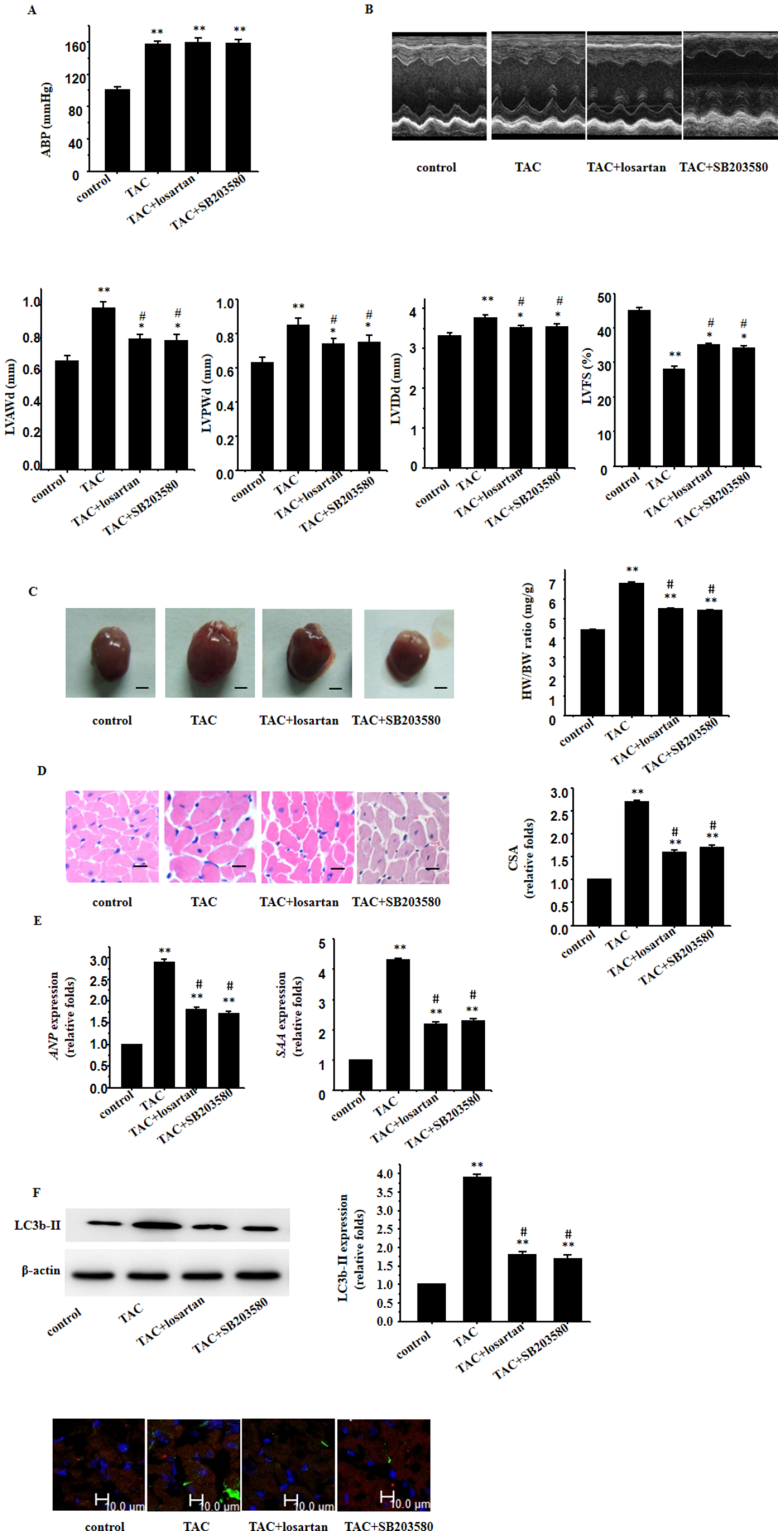
Involvement of cardiomyocyte autophagy in pressure overload-induced cardiac hypertrophy and dysfunction. **A**: BP recordings; Representative recording was shown from 5 mice; **B**: Echocardiographic analysis with representative M-mode tracings from 5 mice. All echocardiograghic data are shown as mean ± S.E.M from 5 mice; LVAWd, LV anterior wall thickness at end-diastole; LVPWd, LV posterior wall thickness at end-diastole; LVIDd, LV internal dimension at end-diastole; LVFS, LV fraction shortening; **C**: Heart morphology and weight; representative global heart photographs of 5 mice (scale bar: 2 mm); heart weigh to body weight radio (HW/BW) measured from 5 mice; **D**: H-E stained LV sections of mice; scale bar: 20 µm; cross sectional area (CSA) of cardiomyocyte measured from 5 sections for one heart and 5 hearts examined; **E**: expression of *Anp and Saa* genes evaluated by the real time RT-PCR. *β-Actin* used as internal control; **F**: Western blot analyses and immunofluorescent staining for expression of LC3b-II proteins using an anti LC3b-II antibody (green); β-actin in whole cell lysate used as the loading control; the hearts also subjected to the immunofluorescent staining for α-MHC (red) and DAPI staining; representative photograms from 5 hearts are shown. All values are expressed as mean ± S.E.M. of 5 mice in all groups; ^*^ *p*<0.05, ^**^ *p*<0.01 vs. control; ^#^ *p*<0.05, ^##^ *p*<0.01 vs. TAC-treated group.

Four weeks later, TAC increased BP (Fig. 4A).Our data demonstrated a severe cardiac hypertrophy following the 4-week TAC, as characterized by an increase in LV wall thickness (Fig. 4B), heart size, heart weight/body weight ratio (Fig. 4C), cross-sectional area of cardiomyocytes (Fig. 4D) and specific gene expressions (Fig. 4E), in conjunction with a lowered LV constriction function (Fig. 4B). Western blot analysis and immunofluorescence for LC3b-II in the heart, revealed overt autophagy in response to pressure overload (Fig. 4F). Losartan and SB 203580 not only rescued against TAC-induced cardiac hypertrophy with an improved cardiac function, but also retarded cardiomyocyte autophagy (Fig. 4A–F).

## Discussion

Autophagy, an evolutionarily conserved process of lysosome-dependent turnover of damaged proteins and organelles [23], plays a pivotal role in the maintenance of cellular environment [10]. Basal autophagy activity helps to maintain cell homeostasis, cardiomyocyte function and ventricular mass. However, excessive autophagy such as in hypertrophied hearts may play a role to prompt transition from ventricular hypertrophy to ventricular failure. It has been reported that pressure overload induced by aortic banding promotes heart failure along with ventricular autophagy in mice [24]. Consistently, upregulation of autophagy was also observed in failing human heart [7]. AT_1_ receptor is considered to mediate Ang II-induced cardiomyocyte autophagy [12]. However, the precise role of AT_1_ receptor and subsequently autophagy in pressure overload-induced transition towards heart failure remains unknown. Data from our study suggested that AT_1_ receptor plays a role in mechanical stress-induced cardiomyocyte autophagy. Data from *in vitro* studies revealed that mechanical stress triggers autophagy in cultured cardiomyocytes. Our findings further revealed that mechanical stress-stimulated cardiomyocyte autophagy was alleviated by Losartan but not AT_2_ receptor antagonist PD123319. These results have favored a role of AT_1_, but not AT_2_ receptor in mechanical stretch-induced cardiomyocyte autophagy.

It is generally perceived that activation of AT_1_ receptor can be turned on by increased secretion of Ang II resulting from various pathological stimuli [25], [26]. Our previous study, however, reported that mechanical stress is capable of activating AT_1_ receptor in the absence of Ang II involvement, thereby inducing cardiac hypertrophy [15]. It is still unknown whether mechanical stress induces autophagy through AT_1_ receptor independent of Ang II. Our results showed that stimulated COS7 cells failed to affect a significant increase in autophagy, while they were induced to develop an increase in autophagy when transfected with AT_1_ receptor. The findings indicated that mechanical stress induces autophagy via AT_1_ receptor independently of Ang II.

The MAPK pathway has been reported to be involved in a number of pathological conditions including cardiac hypertrophy [27], [28], [29]. Our further investigation on autophagy signaling pathway, induced by mechanical stress, revealed that phosphorylation of ERK, JNK and p38MAP kinase was significantly increased following mechanical stress. Employment of pharmacological agents found out that induced autophagic responses were completely inhibited by p38 inhibitor but not ERK or JNK inhibitor. These results suggested that only p38MAP kinase contributes to mechanical stretch-induced cardiomyocyte autophagy even though ERKs, JNK and p38MAP kinase were involved in mechanical stretch-induced cardiomyocyte hypertrophy.

Autophagy has been identified as a well-established mediator of heart failure [24], [30]. Using an established model of aortic constriction [16], we detected a significant activation of autophagy in cardiomyocytes from TAC-operated mice, Losartan and SB203580 attenuated pressure overload-induced cardiomyocyte autophagy along with improved cardiac function, suggesting a possible role of autophagy activation in the development of cardiac remodeling and dysfunction. These findings received support from our *in vitro* study, consolidating the notion that sustained pressure overload may facilitate cardiomyocyte autophagy accompanied with heart failure. It is plausible to speculate a role of AT_1_ receptor and p38MAP kinase in pressure overload-induced autophagy. Our studies further revealed that AT_1_ receptor blocker and p38MAP kinase inhibitor are capable of preventing cardiac remodeling and autophagy in response to chronic pressure overload.

In summary, data from our study revealed that mechanical stress triggers cardiomyocyte autophagy likely through AT_1_ receptor-mediated p38MAP kinase independent of Ang II. Further investigation is warranted to better understand the molecular mechanism of AT_1_ receptor signaling during the pressure overload process.
